# No association between single nucleotide polymorphisms in pre-mirnas and the risk of gastric cancer in Chinese population

**Published:** 2014-02

**Authors:** Jia-Yuan Pu, Wei Dong, Lin Zhang, Wei-Bo Liang, Yan Yang, Mei-Li Lv

**Affiliations:** 1 Department of Respiratory, The First Hospital, Lanzhou University, Lanzhou 730000, China; 2 Department of Immunology, West China School of Preclinical and Forensic Medicine, Sichuan University, Chengdu 610041, China; 3 Department of Forensic Biology, West China School of Preclinical and Forensic Medicine, Sichuan University, Chengdu 610041, China; 4 Department of Obstetrics and Gynecology, West China Second University Hospital, Sichuan University, Chengdu 610041, China

**Keywords:** Pre-miRNA, Polymorphism, rs3746444, rs2910164, rs2292832, rs11614913, Single nucleotide, Stomach neoplasms

## Abstract

***Objective(s):*** Accumulating evidence has demonstrated that miRNAs contribute to various genetic and epigenetic modifications in the pathogenesis of gastric cancer (GC). Recent studies focused on the four single nucleotide polymorphisms (SNPs) of pre-miRNAs including rs11614913, rs3746444, rs2910164, and rs2292832. It was suggested that these four SNPs were significantly associated with the risk of GC and were described as candidate susceptibility factors. However, the previous results show conflicting findings. The aim of this study was to investigate whether these four SNPs are associated with GC in Chinese Han population.

***Materials and Methods:*** Genotype frequencies of these four SNPs of pre-miRNAs in 220 GC patients and 530 control subjects were performed using a PCR-RFLP assay.

***Results:*** No significant differences in genotype and allelic distribution were found in these four SNPs between GC and control subjects in the Chinese Han population. However, we found that the allelic frequency distributions of control subjects in these four SNPs were significantly different from those of the Japanese and the Koreans (All the *P*<0.05).

***Conclusion:*** The four SNPs did not show any significant correlation with the development of GC in the Chinese Han population, based on the current study.

## Introduction

Gastric cancer (GC), the second leading cause of cancer death worldwide, is also the most frequent malignancy of gastrointestinal tract in the East Asian populations (1, 2). Despite continuous progress in improving the diagnosis and treatment, the disease tends to be detected after its invasion of gastric mucosa and the five-year survival rate is less than 20%-25% in patients diagnosed in this late stage (3). The reason lies in the absence of any specific symptoms in early stages and the typical triad of anemia, weight loss and refusal of meat-based foods is seen only in advanced stages. Gastrectomy is the main effective therapeutic approach with limited value, and many studies have tried to find new solutions to resolve this problem. A recent study suggested that the saffron (*Crocus sativus* L.) aqueous extract could inhibit the progression of 1-Methyl-3-nitro-1-nitrosoguanidine-induced gastric cancer in rat, in a dose dependent manner (4). However, a worldwide consensus on the standard management of this disease has not been established so far. 

GC is a complex disease initiated by interaction of the *Helicobacter pylori* infection, environmental and genetic factors, in a multi-step carcinogenetic progress, while the definite etiology for GC is still unclear (5). The most common risk factor is infection with* H. pylori, *which is strongly associated with GC and influenced by some amino acids and sugars (6). It is estimated that two thirds to three quarters of GC worldwide are correlated with* H. pylori* infection (7). Accumulating evidence has determined that various genetic and epigenetic modifications of cell cycle regulator genes, oncogenes, tumor suppressor genes, signaling proteins, and miRNA profile are also potentially involved (5, 8).

Environmental factors also contribute to GC significantly, and tobacco smoking, high consumption of preserved, salted and smoked foods, nitrates as well as carcinogens such as N-nitroso compounds and benzopyrene probably increase GC risk (9, 10). Therefore, *H. pylori* infection interacts with genetic background of susceptibility and environmental factors, finally leading to the development of GC. 

MicroRNAs (miRNAs) are endogenous, small non-coding and single-strand RNA molecules approximately 22 nt. long, which can interact with messenger RNAs (mRNAs) by binding to 3 un-translated regions (UTRs), resulting in the repression of translation, mRNA degradation, and gene silencing (11). At present, studies suggest that miRNAs participate in various biological processes including chromosome architecture, cell proliferation, apoptosis, stress resistance, fat metabolism, embryonic development, and stem cell maintenance (12, 13). Moreover, studies have revealed that miRNAs play important roles in the etiology, progression and prognosis of cancers (14). Therefore, a strong link between altered miRNAs and cancer risk has been established, although the detailed processes still remain elusive. 

Genetic polymorphisms have been shown to be associated with various diseases, such as primary type hypolactasia (15), varicocele-associated infertility (16), and Alzheimer's disease (17). Single-nucleotide polymorphisms (SNPs), which are the most frequent types of variation in the human genome, could provide powerful tools for a variety of medical genetic studies (18). Pre-miRNA is a hairpin structure formed by the full-length miRNA genes’ transcripts (pri-miRNAs). SNPs or mutations in miRNA sequence may affect both the binding to target mRNAs and the pre-miRNA maturation process (19), and even small variations may have an effect on thousands of target mRNAs and result in diverse functional consequences (20). SNPs in pre-miRNA sequences, therefore, may also be functional and represent ideal biomarkers of some diseases.

Moreover, SNPs in susceptibility genes were indicated to contribute to different cancers (-), and SNPs in pre-miRNA have also been shown to be marked as novel genetic variations which may modify the cancer susceptibilities**. **Particularly, the association between the four SNPs (rs11614913, rs3746444, rs2910164, and rs2292832) and digestive diseases becomes the focus of numerous studies, which are located at the pre-miRNA regions of miR-196a2, miR-499, miR-146a, and miR-149, respectively (26). The AG genotype of rs3746444 in the miR-499 has been shown to be significantly associated with the risk of ulcerative colitis (27). Several studies have also reported the positive association between the rs11614913 and rs2910164 polymorphisms and increased susceptibility to digestive system cancers in Asians (28, 29). In addition, genetic polymorphism of miR-196a2 was shown to be significantly associated with increased risk of GC by interfering with its normal binding with target mRNA such as homeobox gene cluster and annexin A1 (30). However, a meta-analysis performed by Xu *et al* indicated that the C allele of the rs2910164 might play an important role in protecting against digestive cancer, and that the rs11614913 contributed to the reduced risk of cancer (31). Therefore, the inconsistent findings require further validation. In the current study, we analyzed the relationship between the four SNPs (rs11614913, rs3746444, rs2910164, and rs2292832), and the susceptibility to GC in the Chinese Han population.

## Materials and Methods

***Subjects***

The study included a population of 220 GC patients and 530 cancer-free controls. All the participants were recruited from West China Hospital, Sichuan University during the period from July 2005 to March 2010, and they were all Chinese Han population living in Sichuan province of southwest China. Exclusion criteria included severe systemic diseases, malignancies in other organs, current or previous genetic diseases. The study was approved by the ethics committee of Sichuan University, and informed consent was obtained from each subject participating in this study. The diagnoses of GC were confirmed by endoscopic biopsy or surgical specimens. Demographic characteristics of the GC patients and control subjects were summarized in Table 1.

***Genotyping***

Blood was taken from all participants by peripheral venous puncture and subsequently stored at -20°C with the anticoagulating agent ethylenedia-minetetraacetic acid (EDTA) until analysis. Genomic DNA of each individual was extracted from 200 l EDTA-added peripheral blood sample with a whole-blood DNA isolation kit (Bioteke Corporation, Beijing, China), following the instructions of the manufacturer. The DNA concentration was measured using a NanoDrop^TM^ 1000 Spectrophotometer (Thermo Fisher Scientific, Copenhagen, Denmark).

**Table 1:** Characteristics of gastric canser patients and control subjects

	Gastric cancer (n=220)	Controls (n=530)	*P*-value
Mean age SD (years)	57.9 11.9	58.6 11.5	0.832
Gender, n (%)			
Male	146 (66.4)	332 (62.6)	0.334
Female	74 (33.6)	198 (37.4)	
Smoking status			
Smoking	138 (62.7)	316 (59.6)	0.428
Non-smoking	82 (37.3)	214 (40.4)	
Alcohol intake			
Alcohol	145 (65.9)	335 (63.2)	0.483
No Alcohol	75 (34.1)	195 (36.8)	

**Table 2:** Genotype distributions for the four SNPs between GC and control subjects

		Gastric cancer		Controls		
SNP	Genotype	n (%)		n (%)	OR (95% CI)	*P*-Value
rs11614913C/T	CC	39 (24.5)		101 (19.8)	Ref	
Gastric Cancer, n=159	CT	95 (59.7)		324 (63.4)	0.759 (0.492-1.173)	0.214
Controls, n=511	TT	25 (15.7)		86 (16.8)	0.753 (0.422-1.343)	0.336
	CT/TT	120 (75.5)		410 (80.2)	Ref	
	CC	39 (24.5)		101 (19.8)	1.319 (0.865-2.012)	0.197
rs3746444C/T	CC	5 (2.6)		17 (3.4)	Ref	
Gastric Cancer, n=196	CT	50 (25.5)		121 (24.0)	1.405 (0.492-4.016)	0.524
Controls, n=504	TT	141 (71.9)		366 (72.6)	1.310 (0.474-3.617)	0.602
	CT/TT	191 (97.4)		487 (96.6)	Ref	
	CC	5 (2.6)		17 (3.4)	0.750 (0.273-2.061)	0.576
rs2910164G/C	GG	36 (18.3)		96 (18.7)	Ref	
Gastric Cancer, n=197	GC	96 (48.7)		274 (53.4)	0.934 (0.597-1.462)	0.766
Controls, n=513	CC	65 (33.0)		143 (27.9)	1.212 (0.748-1.946)	0.434
	GG/GC	132 (67.0)		370 (72.1)	Ref	
	CC	65 (33.0)		143 (27.9)	1.274 (0.894-1.816)	0.180
rs2292832C/T	CC	22 (11.8)		48 (10.5)	Ref	
Gastric Cancer, n=187	CT	31 (16.6)		103 (22.4)	0.657 (0.345-1.251)	0.200
Controls, n=459	TT	134 (71.7)		308 (67.1)	0.949 (0.551-1.635)	0.851
	CC/CT	53 (28.3)		151 (32.9)	Ref	
	TT	134 (71.7)		308 (67.1)	1.240 (0.854-1.800)	0.259

SNP: single-nucleotide polymorphism; OR: odds ratio

The PCR-RFLP analysis was used to genotype the polymorphisms of these four SNPs. PCR were carried out in a total volume of 25 μl, including 50 ng genomic DNA, 2.5 μl 10×PCR buffer, 1.5 mmol/l MgCl_2_, 0.2 mmol/l dNTPs, 0.5μmol/L each primer and 0.3 U Taq DNA polymerase (Bioteke Corporation, Beijing, China), respectively. After PCR amplifications, products were digested with corresponding restriction enzymes overnight and separated by a 6% polyacrylamide gel, followed by staining with 1.5 g/l argent nitrate. Details of the primer sequences, reaction conditions, and restriction enzymes used in this study have been published previously (26, 32). About 10% of the samples were randomly selected to perform the DNA sequencing assays and the results were 100% concordant.

***Statistical analysis***

The SPSS 13.0 software package was used for statistical analysis. Hardy-Weinberg equilibrium, genotype and allele frequencies of the four SNPs were compared between GC and control subjects using the Chi-square test and Fisher’s exact test when appropriate. Results were recorded as the odds ratio (OR) and 95% confidence intervals (CI) to assess the relative risk conferred by a particular allele and genotype. The wild-type genotype/allele served as a reference category. Two-tailed *<* 0.05 was considered statistically significant.

## Results

Demographic characteristics such as age, gender distribution, smoking and alcohol consumption were not significantly different between GC and controls, suggesting that subjects matching based on these variables were adequate (Table 1). 

The number of samples was different for each of the polymorphisms in our study because a few samples failed to genotype at a given locus even after repeating three times.

**Table 3:** Allelic distributions for the four SNPs between gastric cancer and control subjects

SNP	Allele	Gastric cancer n (%)	Controls n (%)	OR(95% CI)	*P*-value
rs11614913C/T					
Gastric Cancer, n=159	C	173 (54.4)	526 (51.5)	Ref	
Controls, n=511	T	145 (45.6)	496 (48.5)	0.889 (0.691-1.144)	0.360
rs3746444C/T					
Gastric Cancer, n=196	C	60 (15.3)	155 (15.4)	Ref	
Controls, n=504	T	332 (84.7)	853 (84.6)	1.005 (0.727-1.390)	0.974
rs2910164G/C					
Gastric Cancer, n=197	G	168 (42.6)	466 (45.4)	Ref	
Controls, n=513	C	226 (57.4)	560 (54.6)	1.119 (0.885-1.415)	0.345
rs2292832C/T					
Gastric Cancer, n=187	C	75 (20.1)	199 (21.7)	Ref	
Controls, n=459	T	299 (79.9)	719 (78.3)	1.103 (0.819-1.486)	0.517

SNP: single-nucleotide polymorphism; OR: odds ratio

**Table 4:** Distributions of the four SNPs allelic frequencies in different populations

	rs11614913				rs3746444				rs2910164				rs2292832		
		Alleles				Alleles				Alleles				Alleles	
	n	C (%)	T (%)		n	C (%)	T (%)		n	G (%)	C (%)		n	C (%)	T (%)
Present study	511	526 (51.5)	496 (48.5)		504	155 (15.4)	853 (84.6)		513	466 (45.4)	560 (54.6)		459	199 (21.7)	719 (78.3)
Japanese ^(^^18^^)^	697	598 (42.9)	796 (57.1)		697	264 (18.9)	1130 (81.1)		697	564 (40.5)	830 (59.5)			-	-
Korean^ (^^27^^)^	447	406 (45.4)	488 (54.6)		447	162 (18.1)	732 (81.9)		447	345 (38.6)	549 (61.4)		447	267 (29.9)	627 (70.1)

The distributions of the rs11614913, rs3746444, rs2910164, and rs2292832 genotypes were in accordance with the Hardy-Weinberg equili-brium among controls. However, no statistically significant differences were found in the genotype or allelic distributions of the four polymorphisms in the pre-miRNAs between GC and control subjects (for rs11614913C/T: OR = 0.889, 95%CI: 0.691-1.144; for rs3746444C/T: OR = 1.005, 95%CI: 0.727-1.390; for rs2910164G/C: OR = 1.119, 95%CI: 0.885-1.415; for rs2292832C/T: OR = 1.103, 95%CI: 0.819-1.486, respectively) (Table 2 and Table 3). 

We conducted the comparison of distributions of the four SNPs allelic frequencies in different populations by comparing our data of control subjects with those in the Japanese and the Koreans reported recently. The allelic frequency distributions of the rs11614913, rs3746444, and rs2910164 in the present study were significantly different from those of the Japanese (All the *P*< 0.05). There were also significant differences in the rs11614913, rs2910164 and rs2292832 allelic distributions between our control subjects and those of the Koreans (All the *P*< 0.05) (Table 4). 

## Discussion

In the present study, we analyzed the potential correlation of the rs11614913, rs3746444, rs2910164, and rs2292832 polymorphisms with GC risk in Chinese people. Our findings revealed that in Chinese Han population, there were no significant differences concerning the allelic and genotype frequencies of the four SNPs between GC and control subjects, which is in accordance with several meta-analysis results (28, 31) and Ahn’s conclusions (33). We also compared the allelic frequencies of the four SNPs polymorphisms between our control groups in the present study and those in the Japanese and the Koreans. Differences in the distributions of allelic frequencies of control subjects between our data and those in Japanese and Korean populations were statistically significant. The allelic frequency distributions of the rs11614913, rs3746444, and rs2910164 in the present study were significantly different from those in the Japanese, as well as the rs11614913, rs2910164 and rs2292832 in the Koreans. This variation in frequency may explain the inconsistency between our negative findings and those of Okubo (29), which suggest genetic background of susceptibility to cancer is different in different populations. 

Previous studies have examined the association between the four SNPs and the risk of GC, and some of the published studies have found positive association between the CC genotype of miR-146a C/G polymorphism and decreased GC risk. For example, Zeng *at al* (34) investigated the miR-146a C/G polymorphism in Chinese population, and found that subjects with GC+GG genotypes showed an increased risk for GC with an OR of 1.58, compared with CC genotype carriers (95% CI: 1.11-2.20, *P* = 0.009). In another analysis of a large cohort of Chinese people, individuals with GG genotype of miR-146a were 1.26-fold more susceptible to GC in comparison to the GC+CC genotypes (95% CI: 1.01-1.56, *P* = 0.038) (35). In the Japanese population, miR-146a was also detected and CC homozygote was demonstrated to be significantly associated with increased risk of GC (29). It was also shown that the rs11614913 SNP in the miR-196a2 was correlated with the degree of *H. pylori* induced mononuclear cell infiltration (29). This conclusion coincides with a case-control study performed by Peng *et al* by showing that the CC genotype of miR-196a2 was more overrepresented in GC patients, compared with TT and CT genotypes (OR= 1.57, 95% CI: 1.03-2.39, *P*=0.038). Stratified analyses further indicated that the CC genotype had a strong association with lymph node metastasis of GC (OR= 2.25, 95% CI: 1.21-4.18, *P*=0.011) (30). However, studies attempting to identify the exact association of the four SNPs in pre-miRNA with GC have yielded conflicting findings. Several meta-analyses confirmed there was no significant association between miR-196a2 polymorphism and increased susceptibility to GC (28, 31). More recently, although suggesting the miR-499A/G AG and AG+GG genotypes were associated with reduced risk of diffuse-type GC, and the miR-149T/C TC and TC+CC genotypes showed lower risk of GC in the male subgroup**,** Ahn *et al* did not find any significant differences in allele and genotype frequencies of the rs11614913, rs3746444, rs2910164, and rs2292832 polymorphisms between GC and controls (33). In summary, the results of these studies remain conflicting rather than conclusive.

To ensure accuracy of the results in the present study, we made some efforts as follows: The non-cancer individual controls were selected strictly from the same geographical regions as the GC patients; There were no statistical differences in the baseline demographic characteristics for the enrolled participants; Ten-percent samples were randomly chosen and direct sequencing was conducted to confirm the results of genotyping by using PCR-RFLP in this study. 

In contrast to a recent study, we failed to detect any significant association between the four SNPs (rs11614913, rs3746444, rs2910164, and rs2292832) and the risk of GC, but this does not necessarily demonstrate that the lack of association in this study rules out the pre-miRNA as a GC candidate gene completely. Like other gene researches, there are some limitations to restrict our conclusions. Although the statistical power in our study was sufficient, the size of patient samples seems to be relatively small if the pathogenic roles of these four SNP polymorphisms are not strong enough. Moreover, as many other complex disorders, GC is thought to be a multi-step process, in which environmental factors interact with a genetic background of susceptibility synergistically. Finally, the ethnic heterogeneity with the collectives may explain the differences of the results between our and Okubo’s study, in which the Japanese population were both genetically and environmentally distinct from our Chinese Han population (29).

## Conclusion

Our study failed to find any association to suggest that the four polymorphisms of pre-miRNA (rs11614913, rs3746444, rs2910164, and rs2292832) confer susceptibility to GC in the Chinese Han population. However, further studies in larger sample sizes and different ethnic populations are needed to clarify the true significance of the association between these four SNPs and GC.
